# Reliability of the heart rate variability threshold during treadmill exercise

**DOI:** 10.1111/cpf.12760

**Published:** 2022-05-19

**Authors:** Trent A. Hargens, Shane Chambers, Nicholas D. Luden, Christopher J. Womack

**Affiliations:** ^1^ Department of Kinesiology, Human Performance Laboratory James Madison University Harrisonburg Virginia USA

**Keywords:** autonomic activity, graded exercise test, root mean square of successive differences of continuous R−R intervals, standard deviation of instantaneous beat intervals, ventilatory threshold, V‐slope method

## Abstract

The heart rate variability threshold (HRVT) is a clinical parameter used to gain insight into autonomic balance. Prior validation of the HRVT has been with cycle ergometry, with no studies examining the viability of treadmill exercise. The purpose of this study was to examine the reliability of the HRVT during treadmill exercise, and to compare the HRVT to the ventilatory threshold (VT). Ten healthy, college‐aged males completed two maximal graded exercise tests on a treadmill. A Polar RS800CX watch was used for heart rate and HRVT data. The HRVT was determined from three HRV variables including the root mean square of successive differences of continuous R−R intervals (RMSSD), the standard deviation of normal R−R intervals (SDNN) and the standard deviation of instantaneous beat intervals (SD1). A metabolic cart was utilized to determine the VT. Results showed no difference between the HRVT (2.4 ± 0.6 and 2.2 ± 0.3 for RMSSD, 2.8 ± 0.5 and 2.7 ± 0.5 for SDNN and 2.4 ± 0.6 and 2.3 ± 0.6 for SD1) or the VT (3.0 ± 0.3 and 3.1 ± 0.3) between trials. When compared to the VT, averaged HRVT values for RMSSD (2.3 ± 0.3) and SD1 (2.3 ± 0.5) were lower than averaged VT (2.8 ± 0.4, *p* < 0.05). The averaged HRVT from SDNN (2.8 ± 0.5) did not differ from the VT. These results suggest that treadmill is a viable mode for HRVT determination, and that HRVT determined by SDNN may be a better comparison to the VT.

## 1 INTRODUCTION

Heart rate variability (HRV) is the variability in the amount of time between successive heartbeats and is used as a biomarker in both athletic and clinical settings. HRV provides insight into an individual's autonomic input, with sympathetic tone leading to decreased HRV, and vagal tone leading to the opposite (Dourado & Guerra, 2013). Accordingly, HRV is generally high at rest with healthy individuals, and decreases with exercise intensity due to vagal withdrawal and sympathetic input (Dourado & Guerra, 2013). As exercise intensity increases and HRV decreases, a threshold is eventually met where interbeat variability no longer decreases with increasing exercise intensity. This is known as the heart rate variability threshold (HRVT) and has been shown to occur at approximately the same intensity as lactate threshold (LT) and ventilatory threshold (VT) (Karapetian et al., 2008). Both the LT and VT are commonly used in clinical and research settings to gain insight into fitness status, as endurance performance is associated with the exercise intensity at which LT and/or VT occurs (Powers et al., 1983). The HRVT is a noninvasive marker which, being linked to the other thresholds previously mentioned, may be a proxy for VT and LT, which require more resources and subject discomfort to obtain. The HRVT is known to be a valuable tool as is corresponds to an exercise intensity at which the transition from moderate to vigorous occurs (Candido et al., 2015).

The process of acquiring HRVT generally requires the subject to undergo a graded maximal exercise test. HRVT has primarily been tested during cycle ergometry (Novelli et al., 2019; Quinart et al., 2014; Sales et al., 2011). However various other modalities such as incremental shuttle tests, and mock‐cross country skiing on a treadmill have been employed (Dourado & Guerra, 2013; Mendia‐Iztueta et al., 2016). To our knowledge, only one prior study used treadmill exercise to assess HRVT, with only a single exercise trial conducted (Paschoal & Fontana, 2011). As such, there is a lack of information regarding the reliability of treadmill use for HRVT detection. Cycle ergometry generally leads to lower $\dot{V}O_{2\max}$ values due to local fatigued induced by decreased muscle mass, and most subjects are more comfortably walking or running on a treadmill compared to cycle ergometry (Hanson et al., 2016; McArdle et al., 1973). It has been suggested that the stabilization points for HRVT determination may be bound by specific limits with regard to the person's $\dot{V}O_{2\max}$, whereas the VT has a greater capacity to respond to training (Grannell & De Vito, 2018). With the differences in $\dot{V}O_{2\max}$ seen between cycle and treadmill exercise, this may impact the relationship between the HRVT and the VT. Therefore, the primary purpose of this study was to examine the within‐subject repeatability of HRVT detection on a treadmill. A secondary purpose was to examine the relationship of the HRVT to the VT, to examine whether they occur at similar exercise intensities.

## 2 METHODS

### 2.1 Participants

Ten, healthy males from a university community were recruited. Active to highly active subjects were recruited (US Department of Health and Human Services, 2018). For this initial study, females were excluded to avoid the impact of menstrual cycle on HRV (Sato et al., 1995). Exclusion criteria included presence of metabolic, pulmonary or cardiovascular disease and regular use of medications that alter HRV (American College of Sports Medicine et al., 2021; Liao et al., 1998; Thayer et al., 2010). Individuals with musculoskeletal disorders were also excluded due to inability to exercise to maximal capacity.

### 2.2 Procedures

All subjects completed a written informed consent before study participation. All research procedures were approved by the Institutional Review Board of James Madison University. Anthropometric data were collected including height and weight. Height was measured via stadiometer. Weight was measured to 0.1 kg on a digital scale with minimal clothing and no shoes. Subjects underwent two maximal graded exercise tests on a treadmill, separated by a minimum of 7 days. A standardized treadmill protocol with 3 min stages was used for each test (Table 1). Gas exchange measurements were obtained continuously via the Parvo Trueone 2400 Metabolic Measurement System (ParvoMedics), which uses a high efficiency mixing chamber. Heart rate and HRV data were monitored and recorded continuously using a Polar RS800CX watch (Polar Electro Inc.).

**Table 1:** Graded exercise test protocol

Stage	Speed (mph)	Grade (%)	Metabolic equivalents (METs)
1	3.0	0	3.3
2	3.5	3.5	5.4
3	3.9	6.5	7.5
4	5.5	0.5	9.6
5	6.0	3.5	11.6
6	6.5	6.0	13.6
7	6.8	9.0	15.6

### 2.3 HRV assessment

R−R interval (RRi) data were obtained with the Polar RS800CX, which has previously been validated against electrocardiographically obtained RRi data (Barbosa et al., 2016; Gamelin et al., 2008; Giles et al., 2016; Hernando et al., 2018). The final 1 min of RRi data obtained during each 3 min stage was used, which presumed that the subjects were in steady‐state exercise (Giles et al., 2016). The RRi data were uploaded using Polar Pro Trainer 5®software. HRV analysis was performed using Kubios Standard software (version 3.1.0.1; Biosignal Analysis and Medical Imaging Group) using moderate artifact filtering (Niskanen et al., 2004).

### 2.4 HRVT and VT determination

Three indexes were utilized to determine HRVT, as previously described (Candido et al., 2015). Briefly, the standard deviation of normal R−R intervals (SDNN), root mean square of successive differences of continuous R−R intervals (RMSSD) and the standard deviation of instantaneous beat intervals (SD1) were utilized. The HRVT was determined to be the first intensity of exercise to present indexes of SDNN, RMSSD and SD1 of less than 3 ms. The HRVT for all three indexes were visually determined by two examiners. If the examiners did not agree on the same intensity, a third examiner was used. If the three examiners did not agree that subject's data were not used in the analysis. Data from two subjects were omitted for RMSSD, data for three were omitted for SD1 while no data were omitted for SDNN.

The VT for each trial was determined using the utilizing the V‐slope method as previously described (Beaver et al., 1986). The VT was determined by the same two examiners as with the HRVT determination, with the same third examiner used if there was not agreement. If the three examiners did not agree that subject's data were not used in the analysis. Data from one subject was omitted for this analysis.

### 2.5 Statistical analysis

Paired *t*‐tests were used to test mean difference in the intensity at which HRVT (as indicated by RMSSD, SD1 and SDNN) and VT occurred. When comparing the three HRVT indexes, averaged values from Trial 1 were compared to averaged values of Trial 2. When comparing HRVT to VT, HRVT values of Trial 1 and 2 were averaged for each respective variable. Bland−Altman plots were utilized to evaluate agreement between HRVT values (RMSSD, SDNN and SD1 derived) between each TM trial, and to evaluate agreement between HRVT and VT.

## 3 RESULTS

### 3.1 Subject characteristics

Subject characteristics are presented in Table 2.

**Table 2:** Subject characteristics (*n* = 10)

	Mean ± SD
Age (year)	20.5 ± 0.7
Height (cm)	160.0 ± 7.1
Weight (kg)	70.0 ± 6.5
BMI (kg/m^2^)	25.8 ± 2.2

Abbreviation: BMI, body mass index.

### 3.2 Reliability of HRVT and VT

**Figure 1:**
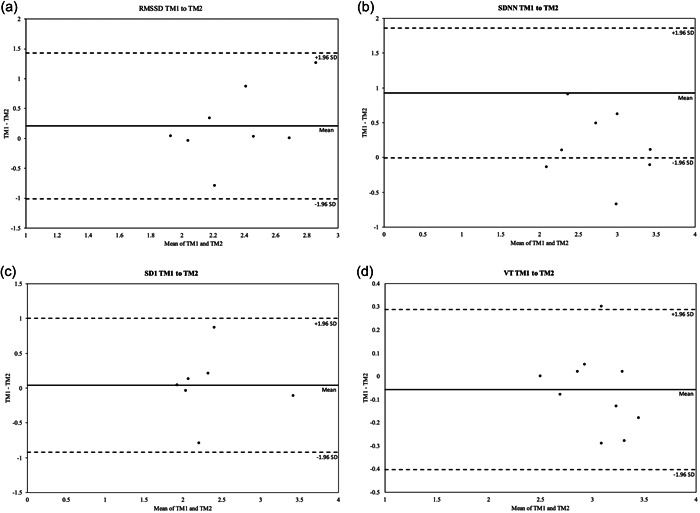
Bland−Altman plots assessing agreement between exercise trials for (a) HRVT_RMSSD_; (b) HRVT_SDNN_; (c) HRVT_SD1_; (d) VT. HRVT_RMSSD_, heart rate variability threshold root mean square of successive differences of continuous R−R intervals; HRVT_SD1_, heart rate variability threshold standard deviation of instantaneous beat intervals; HRVT_SDNN_, heart rate variability threshold standard deviation of normal R−R intervals; VT, ventilatory threshold.

Maximal testing information, HRVT and VT data are presented in Table 3. Maximal heart rate (HR_max_) was significantly lower in Trial 2. There were no differences in $\dot{V}O_{2\max}$, V_E_/VCO_2_ slope or respiratory exchange ratio between trials. No significant differences were found between the VO_2_ at which the HRVT occurred for RMSSD, SDNN or SD1 between trials. When expressed as a percentage of maximal oxygen consumption ($\dot{V}O_{2\max}$), no differences were observed in HRVT between trials. No significant difference was found in intensity at which the VT occurred between trials, when expressed as absolute $\dot{V}O_{2\max}$ or as a percentage of $\dot{V}O_{2\max}$. Bland−Altman analysis revealed good agreement between trials for the HRVT derived from RMSSD (Figure 1a), SDNN (Figure 1b), SD1 (Figure 1c) and VT (Figure 1d).

**Table 3:** Maximal exercise test data

	Trial 1	Trial 2	*p* Value
HR_max_ (beats min^−1^)	191.1 ± 8.7	187.8 ± 9.3	0.03
$\dot{V}O_{2\max}$ (ml kg^−1^ min^−1^)	53.3 ± 6.2	54.1 ± 6.4	0.24
V_E_/VCO_2_ slope	29.3 ± 4.6	29.0 ± 3.9	0.56
RER_max_	1.1 ± 0.04	1.1 ± 0.05	0.32
HRVT_RMSSD_ (L min^−1^)	2.4 ± 0.6	2.2 ± 0.3	0.37
HRVT_SDNN_ (L min^−1^)	2.8 ± 0.5	2.7 ± 0.5	0.55
HRVT_SD1_ (L min^−1^)	2.4 ± 0.6	2.3 ± 0.6	0.83
HRVT_RMSSD_ (% max)	58.5 ± 10.3	53.5 ± 8.6	0.30
HRVT_SDNN_ (% max)	69.0 ± 9.8	65.9 ± 7.4	0.48
HRVT_SD1_ (% max)	55.5 ± 9.8	53.0 ± 9.1	0.72
VT (L min^−1^)	3.0 ± 0.3	3.1 ± 0.3	0.33
VT (% max)	68.0 ± 18.0	69.3 ± 14.4	0.86

*Note*: Data are presented as means ± SD.

Abbreviations: HR_max_, maximal heart rate; HRVT_RMSSD_, heart rate variability threshold root mean square of successive differences of continuous R−R intervals; HRVT_SD1_, heart rate variability threshold standard deviation of instantaneous beat intervals; HRVT_SDNN_, heart rate variability threshold standard deviation of normal R−R intervals; RER_max_, respiratory exchange ratio; VE/VCO_2_ slope, minute ventilation/carbon dioxide production slope; $\dot{V}O_{2\max}$, maximal oxygen consumption; VT, ventilatory threshold.

### 3.3 HRVT comparison

**Figure 2:**
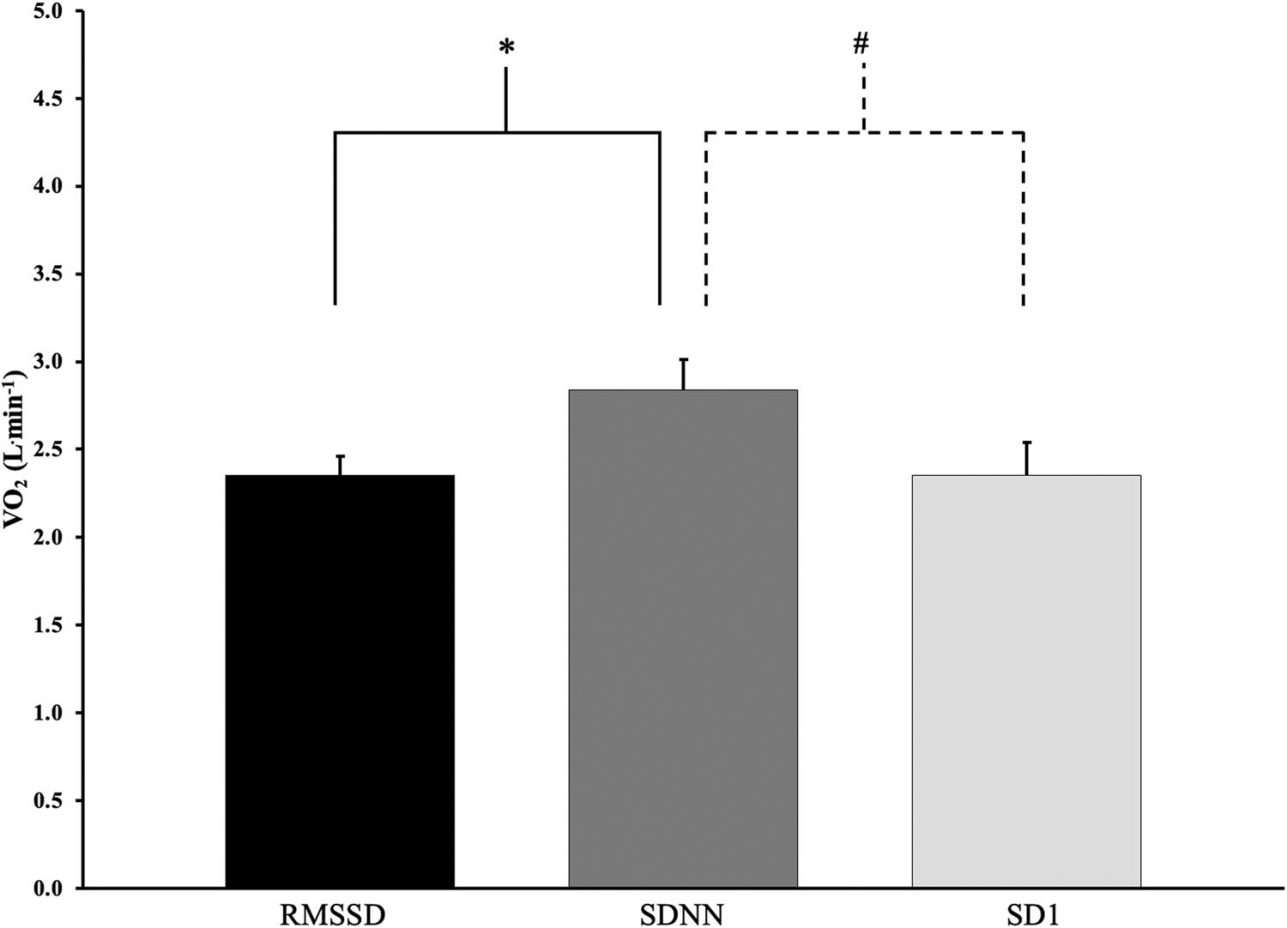
Comparison of the oxygen consumption (L min^−1^) at which the HRVT was identified for three heart rate variability indexes. *p = 0.002. ^**#**^ *p* = 0.02. HSRT, heart rate variability threshold; RMSSD, root mean square of successive differences of continuous R−R intervals; SD1, standard deviation of instantaneous beat intervals; SDNN, standard deviation of normal R−R intervals.

When average HRVT values for each of the three methods were compared, there was no difference in the $\dot{V}O_{2\max}$ at which the HRVT occurred for RMSSD and SD1 (Figure 2). However, the HRVT as determined by SDNN occurred at a significantly higher $\dot{V}O_{2\max}$ when compared to RMSSD and SD1 (Figure 2).

### 3.4 HRVT to VT comparison

**Figure 3:**
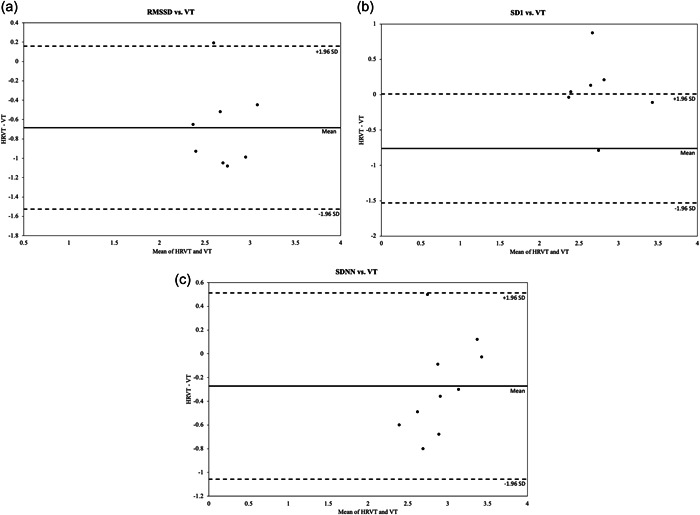
Bland−Altman plots assessing agreement between HRVT and VT. (a) RMSSD; (b) SD1; (c) SDNN. HRVT, heart rate variability threshold; RMSSD, root mean square of successive differences of continuous R−R intervals; SD1, standard deviation of instantaneous beat intervals; SDNN, standard deviation of normal R−R intervals; VT, ventilatory threshold.

Data comparing the occurrence of the HRVT for all three methods and the VT are presented in Table 3. There was no difference at the $\dot{V}O_{2\max}$ in which the VT and SDNN derived HRVT occurred. However, the HRVT derived from the RMSSD and SD1 occurred at a significantly lower $\dot{V}O_{2\max}$ than the VT. Bland−Altman analysis revealed similar findings in that the RMSSD (Figure 3a) and SD1 (Figure 3b) comparisons to VT did not show agreement (*p* = 0.003 and 0.002 for RMSSD and SD1, respectively), while the SDNN to VT comparison (Figure 3c) did show agreement (*p* = 0.06) (Table 4).

**Table 4:** HRVT and VT comparison

	Mean value of two trials
HRVT_RMSSD_ (L min^−1^)	2.3 ± 0.3*
HRVT_SDNN_ (L min^−1^)	2.8 ± 0.5
HRVT_SD1_ (L min^−1^)	2.3 ± 0.5*
VT (L min^−1^)	2.8 ± 0.4

*Note*: Data are presented as means ± SD.

Abbreviations: HRVT_RMSSD_, heart rate variability threshold root mean square of successive differences of continuous R−R intervals; HRVT_SD1_, heart rate variability threshold standard deviation of instantaneous beat intervals; HRVT_SDNN_, heart rate variability threshold standard deviation of normal R−R intervals; VT, ventilatory threshold.

* *p* < 0.05 compared to VT.

## 4 DISCUSSION

The primary findings of this study show that the HRVT derived via treadmill exercise is a reliable measure across multiple exercise trials. This study is the first study to examine the HRVT during treadmill exercise, a much more common mode of exercise for conducting maximal graded exercise tests. A secondary finding of the study is that HRVT derived via SDNN occurred at a similar intensity to the occurrence of the VT. However, HRVT derived from other means (RMSSD and SD1) occurred at a lower intensity compared to the VT, which is in contrast to other published works (Anosov et al., 2000; Cunha et al., 2014; Karapetian et al., 2008).

Prior research into the HRVT has shown that it can be reliably determined across multiple exercise trials, with these findings limited to cycle ergometry (Candido et al., 2015; Novelli et al., 2019). Candido et al. (2015) conducted six separate exercise trials and found no different in the intensity at which the HRVT occurred. They used visual and mathematically determined HRVT, using RMSSD, SDNN and SD1 for their HRVT determination, and used workload (in Watts) as their intensity variable (Candido et al., 2015). Novelli et al. (2019) also found no difference in the occurrence of the HRVT, expressed as Watts and relative oxygen consumption (ml kg^−1^ min^−1^), between two exercise trials. They used visual inspection to determine HRVT utilizing RMSSD and SD1 criteria, and only used RMSSD and SD1 criteria for HRVT determination. Whereas our study and the study conducted by Candido et al. (2015) also used SDNN derived HRVT.

Several studies have examined the relationship between the HRVT and the VT, and have demonstrated the potential utility of the HRVT as a noninvasive and more practical means of estimating the ventilatory and LTs, thereby making it a valuable tool in determining the transition between moderate and heavy exercise domains (Amann et al., 2006; Candido et al., 2015; Dourado & Guerra, 2013; Grannell & De Vito, 2018; Iannetta et al., 2020; Liao et al., 1998; Novelli et al., 2019; Queiroz et al., 2018; Quinart et al., 2014; Sales et al., 2011; Shiraishi et al., 2018). Specifically, there was good agreement between the intensity at which the VT occurs and the HRVT. In addition to all having implemented cycle exercise, these studies have been conducted on a wide range of subjects, including those with type 2 diabetes (Sales et al., 2011), obese adolescents (Queiroz et al., 2018; Vasconcellos et al., 2015), older adults (Dourado & Guerra, 2013), younger adult men and women (Candido et al., 2015; Grannell & De Vito, 2018; Queiroz et al., 2018; Shiraishi et al., 2018) and untrained men and women (Novelli et al., 2019). These studies varied slightly in how HRVT was determined (visually vs. mathematically), and in the criteria for HRVT. A majority of studies utilized RMSSD and/or SD1 data as their basis for HRVT determination. One study utilized frequency domain HRV data for HRVT determination, showing good agreement between the HRVT and the VT (Shiraishi et al., 2018). Only one study utilized SDNN data, and showed that HRVT derived from SDNN, RMSSD and SD1 occurred at similar intensities to the VT (Candido et al., 2015). In contrast to previous, the current study is the only study to utilize treadmill exercise while assessing the reliability of the HRVT, and to compare the HRVT to the VT. Additionally, the current study is only the second study to utilize SDNN‐derived HRVT in comparison to the VT (Candido et al., 2015).

The current study contrasts with most of the prior research in that we observed that only SDNN‐derived HRVT agree with the VT. Both RMSSD and SD1‐derived HRVT did not agree, and occurred at a lower intensity than that of the VT. For RMSSD, the HRVT occurred at an average of 56% of $\dot{V}O_{2\max}$, and for SD1, the average was 54% of $\dot{V}O_{2\max}$. In contrast, the HRVT derived from SDNN occurred at an average of 67% of $\dot{V}O_{2\max}$ and the VT occurred at an average of 69%. Currently, one other study similarly showed that the HRVT and VT did not agree (Grannell & De Vito, 2018). Grannell et al. (2018) utilized a cycle ergometer as their exercise modality and found that HRVT, derived from SD1 data, did not occur at the same intensity as VT. Additionally, they found the HRVT to occur at a mean value of 52% of $\dot{V}O_{2\max}$, which is similar to the current study. In their sample, age and cardiorespiratory fitness level were similar to the current study (Grannell & De Vito, 2018).

Research into the occurrence of the VT has been present since first identified by Beaver et al. (1986). As such, studies into the occurrence of the VT across different modes of exercise have been extensively studied, with differing results based on the population studied. Studies that have compared the occurrence of the VT between walking/treadmill exercise and cycle exercise have shown different findings based on the overall fitness level of those studied. In a more sedentary, clinical population, it has been reported that the VT is consistent across walking and cycling exercise (Hansen et al., 2007). In contrast, in subjects who have higher cardiorespiratory fitness, it has been reported that the VT occurs at a higher intensity with walking/running exercise compared to cycle exercise (Hansen et al., 2016; Kohrt et al., 1989; Medelli et al., 1993; Schneider et al., 1990; Schneider & Pollack, 1991). With an average $\dot{V}O_{2\max}$ across both exercise trials of 53.7 ml kg^−1^ min^−1^ and a mean age of 20.5 years, these subjects would be considered ‘superior’ and above the 75th percentile for their age and sex with regards to their cardiorespiratory fitness (American College of Sports Medicine et al., 2021; Vainshelboim et al., 2020). While the current study did not utilize cycle ergometry, the occurrence of the VT on treadmill was close to 70% of $\dot{V}O_{2\max}$, which is similar to the findings of studies done in fit subjects. Results from the current study extend those findings to treadmill exercise, a much more common mode of conducting graded exercise tests.

The reasons for the disconnect between the RMSSD and SD1‐derived HRVT values and the VT are unclear. RMSSD is the primary time‐domain measure used to estimate the vagally mediated changes in HRV, whereas SDNN reflect both sympathetic and parasympathetic activity (Shaffer & Ginsberg, 2017). Additionally, the nonlinear SD1 is identical to RMSSD in reflecting parasympathetic activity (Ciccone et al., 2017; Shaffer & Ginsberg, 2017). Therefore, a reduction in HRV during exercise, as measured by RMSSD or SD1, is more dependent on parasympathetic withdrawal than increased sympathetic nervous system drive. Traditional thinking stated that during progressive exercise, the initial heart rate increase is mediated primarily by parasympathetic withdrawal, up to a heart rate of approximately 100 beats min^−1^ (Rowell, 1993). Beyond that, the slower acting sympathetic activation becomes the primary factor in increasing the heart rate, and subsequently a reduction in HRV. However, more recently is has been suggested that complete parasympathetic withdrawal does not occur during exercise, but rather both sympathetic and parasympathetic contribute equally to the HR change during exercise up to approximately 140 beats min^−1^ (White & Raven, 2014). Beyond that, the sympathetic system becomes dominant, but parasympathetic influence remains until maximal exercise (White & Raven, 2014). White and Raven propose that as exercise is initiated, the arterial baroreflex is ‘reset’, which results in a decrease in parasympathetic activity and a slight decrease in sympathetic activity due to increased venous return with exercise (White & Raven, 2014). As exercise intensity increases, increases in arterial baroreflex resetting augments the sympathetic increases in HR and depresses parasympathetic, resulting in greater sympathetic influence at higher intensities (White & Raven, 2014). It is possible that the HRVT, as derived by RMSSD and SD1, are occurring at a lower intensity when compared to SDNN‐derived HRVT due to greater parasympathetic influence, whereas SDNN reflects sympathetic activation as well. This could also explain why the VT and SDNN‐derived HRVT are in greater agreement. Due to the slower nature of sympathetic activation, when compared to parasympathetic activation, the accumulation of lactate and subsequent ventilatory response are occurring at a slightly higher intensity, better reflected by SDNN.

### 4.1 Limitations

This study is limited by a small sample size. However, previous published research has utilized similar methods and sample sizes to the current study (Grannell & De Vito, 2018). Additionally, this study relied on visual determination of HRVT and VT. These methods, however, remain the most common method for threshold determination, as more objective means of threshold determination have yet to be identified (Candido et al., 2015). Finally, we did not include females in the current sample. This was done to minimize the impact that menstrual cycle has on measures of HRV. Future studies must include females, with proper monitoring of menstrual cycle to control for the impact it has on HRV (Liao et al., 1998; Thayer et al., 2010).

## 5 CONCLUSION

Results of the current study showed that HRVT is a reliable measure when obtained with treadmill exercise in young, healthy males. Additionally, we found that the HRVT, when derived from SDNN, did not differ in the intensity at which they occurred when compared to the VT, and showed good agreement. Given that the VT has been used extensively as a marker of aerobic fitness, as well as an important intensity for exercise training in healthy and clinical populations, the more cost efficient and easier to obtain measures of HRV and HRVT, could have utility to a broad spectrum of individuals (Mazaheri et al., 2021; Tan et al., 2017). In contrast, the HRVT, derived from RMSSD or SD1, occurred at a lower intensity than the VT. This may reflect differences in the HRV methods, with respect to autonomic contribution to the variables. Further research is needed to assess this relationship, as well as assessing the reliability within and across exercise modes.

## CONFLICTS OF INTEREST

The authors declare no conflicts of interest.

## Data Availability

Data are available on a reasonable request.
